# Regional blocks for pain control at the end of life in pediatric oncology

**DOI:** 10.3389/fpain.2023.1127800

**Published:** 2023-03-21

**Authors:** Andrea Cuviello, Ashley Cianchini de la Sota, Justin Baker, Doralina Anghelescu

**Affiliations:** ^1^ Department of Oncology, St. Jude Children's Research Hospital, Memphis, TN, United States; ^2^ Department of Medicine, University of Puerto Rico, San Juan, Puerto Rico

**Keywords:** palliative care, pain management, end of life, pediatric oncology, continuous nerve block, single-shot nerve block

## Abstract

**Background:**

Pain management at the end of life is a fundamental aspect of care and can improve patients' quality of life. Interventional approaches may be underutilized for pediatric cancer patients.

**Objective:**

To describe a single institution's 10 years of experience with regional pain management at the end of life in pediatric oncology.

**Methods:**

A retrospective cohort study of 27 patients with pediatric cancer who died between April 2011 and December 2021 and received continuous nerve block (CNB) catheters or single-shot nerve blocks (SSBs) during their last three months of life. The type of blocks, analgesic efficacy, and palliative care involvement were evaluated.

**Results:**

Twenty-two patients (81.5%) had solid tumor diagnoses, including carcinomas, sarcomas, and neuroblastoma. Most (59%) patients received CNB catheters, and 12 patients (44%) received SSBs for pain control. The mean pain score decreases for CNB catheters and SSBs after interventions were −2.5 and −2.8, respectively, on an 11-point scale. Decreases in opioid patient-controlled analgesia dosing requirements were noted in 56% of patients with CNB catheters; likewise, in 25% of patients with SSBs at 24 h and in 8% at 5 days after interventions. Nearly all patients had PC involvement and received care from pain specialists (96% and 93%, respectively). Twenty-three (85%) had physician orders for scope of treatment orders completed before death.

**Conclusion:**

Regional pain control interventions can be effective and safe for relieving regional pain and suffering in dying children and young adults. The collaboration between palliative care and pain management specialists at the end of life can help alleviate suffering and improve quality of life.

## Introduction

Pain management during end-of-life (EoL) care remains important to optimize a patient's overall quality of life (QoL). Up to 50% of patients undergoing cancer treatment experience pain secondary to their disease. This increases to 76% during the EoL period and can be as high as 90% for pediatric patients with advanced cancer (1, 2). In a study of 185 pediatric patients, pain was reported in 91.5%, being highest in those patients with solid tumors (2, 3). Pain can significantly affect both patient and family QoL, and studies show that relief of suffering, including pain symptoms, is at the forefront of patient, family, and healthcare providers' goals (4). Using a holistic approach, palliative care (PC) is a subspecialty that can provide symptom relief and improve a patient's QoL (5, 6). In larger institutions, PC physicians often work with the primary oncology team and others, including pain medicine specialists and integrative medicine, to provide targeted pain relief interventions (7), with a focus on improving EoL care.

Strategies to enhance QoL and relieve suffering due to pain are multimodal and multidisciplinary and include the following: (1) pharmacological therapy including but not limited to NSAIDs, acetaminophen, opioids, methadone, and gabapentinoids, (2) physical and occupational therapy, (3) advanced infusions such as low-dose ketamine or lidocaine infusions, and (4) interventional approaches such as peripheral nerve blocks and central neuraxial blocks (intrathecal or epidural) (8). Additionally, integrative medicine approaches including acupuncture and massage are being incorporated more frequently for pain and other distressing symptoms (9, 10). Occasionally, traditional symptom management strategies are insufficient to alleviate suffering, so consideration of palliative sedation therapy may be needed (11).

Morphine is the most commonly used drug for pain control in the EoL period (reported in 60%–90% of patients) (12); opioids and other pharmacological therapies can cause significant side effects such as constipation, sedation, and nausea (13), increasing patient distress. Therefore, care providers must optimize pain management regimens and incorporate interventional approaches, when appropriate, to reduce pain and systemic opioid exposure and related side effects. Regional pain interventions such as continuous nerve block (CNB) catheters have been used to decrease pain in both pediatric (8) and adult patients (14); however, the efficacy of single-shot nerve blocks (SSBs) and the use of interventional pain management modalities during EoL care in pediatric cancer patients are not well described. Here, we evaluate the use of regional pain control interventions for children with cancer-related pain during the EoL period.

## Patients and methods

In this Institutional Review Board–exempt retrospective review, we evaluated pediatric patients from a single academic institution who were treated with CNB catheters or SSBs as part of regional pain control regimens during the EoL period [defined as the three months before the documented date of death (DOD)]. The study institution is a 78-bed facility that cares for over 500 new pediatric patients with oncology diagnoses each year, comprising patients from the Mid-South as well as national and international patient referrals. Pediatric patients were defined as those who received their primary diagnosis before the age of 21 years or received a diagnosis of pediatric primary cancer. Patient data were obtained from the electronic medical record database PowerChart P134 from CernerWorks. The study review period included patients who died between April 2011 and December 2021, a timeframe during which patients would have all pertinent data for this study placed into the electronic medical record system. Demographic information [age, primary diagnosis, DOD, and location of death (LOD)] was collected. The following pain characteristics were collected at the interventional procedure for pain: (1) location of pain; (2) type of pain (nociceptive, neuropathic, visceral, or somatic); (3) medications for pain management (opioids, anticonvulsant, non-steroidal anti-inflammatory drugs, tricyclic antidepressants, acetaminophen, methadone, ketamine, and corticosteroids); and (4) pain scores (PS) collected 24 h before and 24 h after the interventional procedure, and, as applicable, 5 days after the procedure. For the PS assessment, age-appropriate pain assessment tools were used, including the FACES Revised pain scale (15), FLACC score pain scale (16), and the numeric rating scale (17). A follow up period of 5 days was chosen to limit data inconsistencies that may arise from patients moving between inpatient and outpatient settings, and therefore possibility of missing data. as well as to mitigate the effects that active dying may have on symptom management needs. PC data collected included the (1) preferred death location (if stated), (2) number of PC team visits, (2) number of pain management specialist visits, (3) physician orders for scope of treatment (POST), and (4) patient lifespan following the regional pain block intervention.

The data collected regarding pain management interventions included intervention type (i.e., epidural catheter, intrathecal catheter, peripheral nerve block catheter, or single-shot nerve block); device location; tunneling technique (i.e., tunneled or not tunneled); local anesthetic and/or ablative agent, concentration, and rate of infusion at day of insertion and by day 5 after insertion (if applicable); duration of the device in relation to the patient's lifespan (measured in days); outpatient status with device; and reason for device removal (if applicable). Potential limiting factors for assessing a patient's candidacy for the interventional pain blocks were collected, specifically the absolute neutrophil count (ANC) (×10^3^·1^−1^) and the platelet count (×10^−1^·1^−1^). Adverse effects following the pain blocks were evaluated.

The change in PS before and 24 h after the pain intervention, as well as the change in opioid patient-controlled analgesia (PCA) requirement before intervention, 24 h post, and at day 5 after intervention were used to analyze the effectiveness of the pain block interventions. Patients who did not receive PCA before or after the intervention were evaluated using only the change in PS before and 24 h after the intervention. When PS were not documented within a 24 h interval, this was counted as missing data. To facilitate comparison of different intravenous opioids used for PCA, the doses were calculated as mean intravenous morphine equivalent (mg·kg^−1^·day^−1^), using the following equianalgesic rations: 100:1 for fentanyl:morphine and 5:1 for hydromorphone:morphine.

## Results

### Patient demographics

Altogether, 2,151 patients died during the study period; 415 of them (19%) engaged with the pain medicine team. Of those who received subspecialty pain care, 27 (6.5%) received interventional pain management procedures during the EoL period and were reviewed for this study; of them, 4 patients had multiple interventional procedures reported. Table 1 summarizes the demographic data collected. Briefly, the mean age was 14 years (range: 2 to 26 years), and the predominant primary diagnosis was osteosarcoma (18.5%, *n* = 5), with most patients (81.5%, *n* = 22) having solid tumor diagnoses. The most common site of pain was the abdominal region (48%, *n* = 13), and most patients (74%, *n* = 20) had a documented nociceptive type of pain.

**Table 1:** Demographic data of the 27 patients who received an interventional pain modality during the end of life.

Case No.	Age	Primary diagnosis	Site of pain	Type of pain
1	20	NCA	Abdomen	–
2	2	HBL	Abdomen	N/V
3	8	LCH	Abdomen	N/V
4	20	ERMS	Pelvis	N/V
5	7	OS	Arm	N
5.1			Leg	N
6	22	HL	Shoulder	N/NP
6.1			Lower Back	N
6.2			Leg	N/NP
7	17	GCT	Abdominopelvic	N/V
8	16	AML	Abdomen	V/N
9	16	OS	Arm	N
9.1				
9.2				
10^*^	22	CCS	Thumb	–
11	5	AML	Abdomen	N/V/S
11.1				
12	4	HBL	Chest	–
13	17	ADCA	Lower Back	S
14	20	DSRCT	Back, Abdomen	N/V
15	10	SYNS	Leg	NP/S
16	19	PNET	Back	–
17	26	ALL	Knee	N
18^*^	15	NBL	Abdomen	N/V/S
19	15	RMS	Perinium	N/NP
20	8	RMS	Abdomen	–
21	22	ES	Lower Back	S
22	20	RMS	Abdomen	–
23^*^	22	OS	Leg	N
24^*^	9	OS	Leg	N/NP
25	10	OS	Leg	N
26^*^	5	RMS	Abdominopelvic	N/V
27	3	W	Abdomen	–

ADCA, Adenocarcinoma; ALL, Acute lymphocytic leukemia; AML, acute monocytic leukemia; CCS, clear-cell sarcoma; DSRCT, Desmoplastic small round cell tumor; ERMS; Embryonal rhabdomyosarcoma; ES, Epithelioid sarcoma; EWS, Ewing sarcoma; GCT, Granulosa cell tumor; HBL, hepatoblastoma; HL, Hodgkin lymphoma; LCH, Langerhans cell histiocytosis; MS, Myeloid sarcoma; NBL, neuroblastoma; NCA, Neuroendocrine carcinoma; OS, Osteosarcoma; Pan, Pancreatic cancer; PNET, Primary central nervous system tumor; Rb, Retinoblastoma; RMC, Renal medullary carcinoma; RMS, Rhabdomyosarcoma; SMCT, Smooth muscle cell tumor; W, Wilms Tumor; N, nociceptive pain; NP, neuropathic pain; V, visceral pain; S, somatic pain.

^*^ Patients received a nerve block catheter or single-shot nerve block to manage pain from a surgical procedure related to their diagnosis. Patients who had more than one regional pain control intervention are identified with decimal points.

### Continuous regional pain control interventions

Most patients (59%, *n* = 16) received CNB catheters, 62% (*n* = 10) of which were epidural catheters (Table 2). Additionally, 19% (*n* = 3) received multiple CNB catheter infusions: one patient had two peripheral nerve block catheters placed on the same day in two different locations, and two patients had dislodged catheters replaced. The average use of a CNB was 6 days (range, 4–15 days) for peripheral nerve block, 16 days (range 5–24 days) for intrathecal catheters, and 11 days (range 2–56 days) for epidural catheters (Table 2). Three-quarters (*n* = 12) of the catheters were placed using a tunneling technique, and 62% (*n* = 10) remained in place on the DOD. Most patients (88%, *n* = 14) using CNB catheters were receiving opioids (excluding methadone) prior to catheter insertion.

**Table 2:** Patients with continuous nerve block catheters for pain management at the end of life.

Case No.	Pain medication regimen at block placement	Device type	Device location	Tunneled (y/n)	Anesthetic agent(s) at insertion	Change in mean pain score at 24 h	Change in PCA at 24 h (mg/h)	Change in PCA at 5 days (mg/h)	Device duration/Patient lifespan (days)	Outpatient with device (y/n)	Reason for removal
2	Op, APAP, NSAID	PNB	ESP	–	Ropivacaine	−2	–	–	4/15	n	Post-operative
5	Op, Me, K, APAP	PNB	Supraclavicular	y	Ropivacaine	4	0	2.5	6/6	n	Death
5.1	Op, Me, K, APAP	PNB	Femoral (L)	y	Ropivacaine	8	0	2.5	6/6	n	Death
6.2	Op, APAP, K, NSAID	IT	L3-L4	y	Bupivacaine/Morphine	−2	−25	–	5/5	n	Death
7	Op	IT	L3-L4	–	Ropivacaine/Fentanyl	−8	–	–	24/24	y	Death
9	Op	PNB	Interscalene	y	Ropivacaine	0	0	–	3/43	n	Dislodged
9.1	Op	PNB	Interscalene	y	Bupivacaine	−1	−5	−7.5	14/43	y	Dislodged
9.2	Op	PNB	Interscalene	y	Ropivacaine	−6	−4	−1.5	21/21	y	Death
11	Op, Gbp, Me	E	T8-T9	n	Ropivacaine	0	0	–	8/56	n	Dislodged
11.1	Op, Gbp, Me	E	T9-T10	y	Bupivacaine	−4	0	–	5/56	n	Dislodged
12	None	E	T6-T7	–	Ropivacaine	1	–	–	4/45	n	Post-operative
13	Op, Me, K, NSAID	IT	L3-L4	–	Bupivacaine/Hydromorphone	−4	0	−75	18/18	n	Death
14	Op, Me	E	T10-T11	y	Bupivacaine	−3	7	−5	56/56	n	Death
15	Op, Gbp, TCA	E	L2-L3	y	Ropivacaine/Fentanyl	−3	−0.6	–	3/37	n	Travel
18^*^^^^	None	E	T12-L1	–	Ropivacaine/Fentanyl	0	–	–	3/3	n	Death
19	Op, TCA, Me	E	L4-L5	y	Ropivacaine	−4	5	10	25/25	n	Death
21	Op, Gbp, NSAID	E	T12-L1	y	Ropivacaine/Fentanyl	−3	−2	8	3/21	n	Dislodged
24^*^	Op, Gbp	E	L4-L5	–	Ropivacaine/Fentanyl	−10	−25	–	4/16	n	Thoracocentesis
25	Op, Gbp, Me, NSAID	E	L2-L3	y	Bupivacaine	−2	0	3	7/7	n	Death
26^*^	Op	E	L1-L2	–	Ropivacaine/Fentanyl	−4	0	0.1	4/42	n	Post-operative

PNB, peripheral nerve block catheter; E, epidural catheter; IT, intrathecal catheter; Op, opioid; Me, methadone; K, ketamine; Gbp, gabapentin; NSAID, non-steroidal anti-inflammatory drugs; TCA, tri-cyclic antidepressants; APAP, acetaminophen; ESP, erector spinae plane block.; [ ], concentration.

^*^ Patients received a nerve block catheter related to pain management for a surgical procedure related to their diagnosis.

^^^ Patient had their catheter turned off for fewer than 24 h for an adverse event.

The mean PS was 5 (range, 0–10) before catheter insertion and 3 (range, 0–10) 24 h after insertion. Nearly all catheter-based interventions (88%, *n* = 14) resulted in decreasing PS, with a mean decrease of 2.15 (Table 2). Three-quarters of patients receiving CNB intervention (*n* = 12) had an opioid PCA as part of their pain management regimen. After CNB intervention, 37% (*n* = 6) had decreased PCA dosing requirements 24 h after catheter placement; 37% had decreased PCA dosing requirements 5 days after catheter placement.

### Single-shot regional pain control interventions

Nearly half (44%, *n* = 12) of patients received a SSB intervention for pain control, mainly celiac plexus blocks (*n* = 7, 58%; Table 3). One patient (patient 6) had both a suprascapular and lumbar plexus SSB performed one week apart to address pain from the primary tumor site and diffuse pain from worsening metastatic disease, respectively. Before intervention, opioids (excluding methadone) were used for pain control by most patients (83%, *n* = 10).

**Table 3:** Patients with single-shot nerve blocks for pain management at the end of life.

Case No.	Pain medication regimen at time of block	Location	Anesthetic agent(s) at insertion	Change in mean pain score at 24 h	Change in PCA at 24 h (mg/h)	Change in PCA at 5 days (mg/h)	Lifespan after intervention (days)
1	Op	Celiac plexus	Ethanol/Bupivacaine/Lidocaine	−8	−5	−5	49
3	Op, APAP	Celiac plexus	Ethanol/Bupivacaine	−4	−0.3	−0.3	39
4	Op, Gbp, Me	Hypogastric plexus	Bupivacaine/Ethanol/Lidocaine	−4	–	–	49
6	Op, Me, Ox, NSAID	Suprascapular	Methylprednisolone/Bupivacaine/Lidocaine	−1	0	10	19
6.1	Op, Me, OxAPAP, Ox, K	Lumbar plexus	Lidocaine	0	0	20	26
8	Me, Gbp, Ox	Celiac plexus	Ethanol/Bupivacaine	−2	–	1	86
10^*^	Op, Me	Supraclavicular	Bupivacaine	0	–	–	32
16	Op, Me, Cs, Bz	Celiac plexus	Ethanol/Bupivacaine	−6	−1.5	–	6
17	None	Femoral (L)	Bupivacaine	1	–	–	50
20	Op, Me	Celiac plexus	Ethanol/Bupivacaine	−4	0	−0.3	17
22	Op, Gbp	Celiac plexus	Ethanol/Bupivacaine	N/A	–	–	37
23^*^	None	Femoral (L)	Bupivacaine	N/A	–	–	71
27	Op	Celiac plexus	Ethanol/Bupivacaine	N/A	–	–	91

Op, opioid; APAP, acetaminophen; Gbp, gabapentin; Me, methadone; N/A, not available; NSAID, non-steroidal anti-inflammatory drugs; OxAPAP, oxycodone/acetaminophen; Ox, oxycodone; Cs, corticosteroids; K, ketamine; Bz, benzodiazepine.

^*^ Patients received a nerve block related to a surgical procedure related to their diagnosis.

Over half of these patients (58%, *n* = 7) had a documented decrease in PS at 24 h after the SSB intervention; the mean change in PS was −2.8, with a median change of −3 (Table 3). Of the 5 patients who were on PCA management before SSB intervention, 25% (*n* = 3) had a decreased PCA requirement at 24 h, and 17% (*n* = 2) had no change in the PCA requirement. Five days after the intervention, 17% (*n* = 2) had decreased PCA requirements, and another 17% had an increased or new PCA requirement (Table 3). Three patients (25%) did not have PS recorded before or after the procedure, so their analgesic efficacy could not be evaluated.

### Palliative care characteristics

Nearly all patients in this study (96%, *n* = 26) had PC involvement, with an average of 40 visits, not limited to the EOL period, throughout their care journey (range, 2–120). Similarly, 93% (*n* = 25) of patients received care from a pain medicine specialist, with an average of 37 visits spanning the course of their clinical care (range, 1–223). Of the patients who received a CNB catheter, 2 (7.4%) used their devices in the outpatient setting. The mean lifespan following regional pain control intervention was 34 days (median, 32 days; range, 3–91).

Most patients (85%, *n* = 23) had POST orders completed before the DOD: 70% (*n* = 16) indicated limited additional interventions, and 22% (*n* = 5) specified comfort measures only (Table 4). Over half (59%, *n* = 16) died in a hospital setting although only 5 of the 11 patients who specified a preferred LOD chose a hospital setting. Of the patients who set a preferred LOD, 64% (*n* = 7) died at their preferred location (Table 4).

**Table 4:** Palliative care characteristics of patients who received regional pain control intervention.

Case No.	Lifespan following intervention (days)	Outpatient with device (y/n)	Death location (preference/actual)	POST	SoMI	No. of QoL visits	No. of pain visits
1	49	n	Not stated/Home	–	–	78	0
2	15	n	Not stated/Hospital	DNR	LAI	27	20
3	39	–	Not stated/Ambulance	–	–	79	7
4	49	–	Not stated/Home	DNR	–	21	1
5	6	n	Hospital/Hospital	DNR	LAI	34	27
5.1	6	n	Hospital/Hospital	DNR	LAI	34	27
6	19	–	Not stated/Hospital	DNR	LAI	52	57
6.1	26	–	Not stated/Hospital	DNR	LAI	52	57
6.2	5	n	Not stated/Hospital	DNR	LAI	52	57
7	24	y	Hospital/Home	DNR	CM	85	14
8	86	–	Outpatient/Outpatient	DNR	LAI	59	30
9	43	n	Not stated/Home	DNR	LAI	29	32
9.1	40	y	Not stated/Home	DNR	LAI	29	32
9.2	29	y	Not stated/Home	DNR	LAI	29	32
10	32	–	Not stated/Hospital	DNR	LAI	62	9
11	56	n	Not stated/Hospital	DNR/DNI	CM	56	102
11.1	48	n	Not stated/Hospital	DNR/DNI	CM	56	102
12	45	n	Not stated/Unknown	–	–	0	5
13	18	n	Not stated/Hospital	DNR/DNI	LAI	120	31
14	56	n	Not stated/Hospital	DNR	LAI	93	223
15	37	n	Not stated/Hospital	DNR	–	4	53
16	6	–	Not stated/Hospital	DNR	CM	18	6
17	50	–	Not stated/Hospital	DNR	CM	2	15
18	3	–	Not stated/Hospital	–	–	3	3
19	25	n	Hospital/Hospital	DNR	LAI	65	150
20	17	–	Not stated/Hospital	DNR	LAI	29	0
21	21	n	Home/Home	DNR/DNI	CM	12	22
22	37	–	Home/Hospital	DNR	LAI	7	0
23	71	–	Home/Home	DNR	LAI	26	13
24	16	n	Home Hospital/Hospital	DNR	LAI	29	17
25	7	n	Hospital/Hospital	DNR/DNI	LAI	33	58
26	42	n	Home/Home	DNR	LAI	14	16
27	91	–	Target House/Home	DNR	LAI	19	11

DNR, do not resuscitate; DNI, do not intubate; SoMI, scope of medical intervention; LAI, limited additional interventions; CM, comfort measures.

### Contraindications and limitations to regional pain control interventions

One patient experienced moderate neutropenia (ANC <1,500 and ≥1,000) and two patients had platelet counts <50k. Four adverse events were reported in our patient cohort: ventricular tachycardia, bleeding, leaking of the catheter, and a temporary discontinuation of catheter infusion due to a surgical procedure. Of note, the reporting of potential complications spans the time period of date of regional pain intervention until the day of death, to ensure potential procedural complications were not inadvertently missed.

## Discussion

This single-institution retrospective study reports on a decade of regional pain management interventions for pediatric oncology patients during the EoL period and represents the largest known cohort to date. Historically, regional pain management interventions have been underutilized during the EoL period because of concerns about procedural complications, such as infection and bleeding, patient distress/discomfort during the procedure, and the time requirements for patient assessment (18). Likewise, our study reports a low utilization rate of interventional approaches in only 6.5% of the patients followed by the pain specialists in this series. Underutilization of interventional approaches for pain is of concern, especially in view of our findings that highlight their analgesic efficacy as reflected by decreased PS and opioid utilization, safety, and benefits of these regional pain management interventions as efforts toward increasing QoL during EoL care for children with cancer. Our findings suggestive of underutilization of interventional pain management in pediatric oncology are consistent with those of a recent review article in pediatric oncology (19), which supported the fact that interventional approaches are typically reserved for refractory pain unresponsive to noninvasive treatment. Similarly, a review of literature from 1980 to 2012 indicated that although regional techniques are usually considered only in the limited context of failure of systemic treatments and/or intolerable medication side effects, their associated risks are often acceptable when the potential benefits are consistent with the overall goals of care (20).

Despite leukemias remaining the most prevalent pediatric cancer type (21), 81% of patients in our study cohort had a solid tumor primary diagnosis. This was unsurprising as our study focused on pain in the EoL period, and patients with solid tumor diagnoses often have an increased tumor burden, contributing to increased pain (3, 22). Solid tumors are thought to release neuroimmune mediators that activate nociceptive nerve terminals, leading to increased nociceptive pain (23). Almost two-thirds of our cohort reported nociceptive pain, as expected given the predominance of solid tumor diagnoses reported. The various locations of reported pain were directly correlated with tumor location, as expected, and influenced the location of CNB or SSB. Regardless of which regional pain control intervention was used, opioids were a mainstay of pharmacological pain regimen. In many patients, opioids were administered as intravenous PCA. This was an expected finding as opioids are frequently prescribed for EoL symptom management and often require massive and rapid dose escalation in this setting (12, 24, 25).

Case reports and case series in adults and pediatrics emphasize that CNB catheters can mitigate pain for patients with advanced cancer pain (8, 14, 26); in our study, CNB catheters were used for most patients. Of these patients, 25% required less opioid medication to achieve pain relief at 24 h and 5 days after placement, and 88% had a lower PS. It may appear from our data that celiac plexus CNB were less effective in this series despite prior case series reporting analgesic efficacy (27). We attribute this potential correlation with cancer progression in the EoL period. CNB can sometimes be used for prolonged periods, with the literature demonstrating duration of use of 81–240 days (8, 28, 29). Our study cohort demonstrated catheter duration of 4–56 days. Additionally, 10 patients kept their catheters in place until the DOD, with two continuing this intervention in the home setting. Together, our findings show that CNB can be important in EoL pain management, can be tolerated for prolonged periods, and may serve patients in the outpatient and home/hospice setting.

Similar to CNB, little has been published about the role of SSBs as pain management for EoL care. One study reported SSB of the brachial plexus to be useful for adults (22), with pain relief sometimes lasting up to 10 months (30). In our patient cohort, nearly half of patients received a SSB, with 58% reporting a decrease in PS intensity. In patients who had an opioid PCA regimen before their SSB, four had a decrease in their opioid PCA dosing requirements after the intervention was performed, suggesting improved pain relief secondary to the SS nerve block. Notably, two patients (16%) reported no change in PS after the procedure, and one patient (8%) reported an increase in PS. We hypothesize that this may be partially due to incomplete optimization of adjuvant pain control such as oral pharmacological regimen or PCA, technical difficulties in performing the blocks, or disease progression and extension outside the dermatomal levels blocked. Additionally, one patient (patient 6) did not have successful pain relief after two SSBs performed in two different anatomical locations due to progressive tumor burden, and thus underwent escalation of their pain management regimen to include a continuous intrathecal catheter, which did result in improved PS and decreased PCA opioid requirements. These findings establish the potential benefits of regional pain control interventions and should encourage physicians to partner with their interdisciplinary colleagues to offer these modalities for relief of suffering whenever appropriate.

Clinicians may avoid regional pain control interventions at the EoL for fear of procedural complications such as bleeding and infection or for worries that it may interfere with patient and family EoL preferences, such as dying at home (8, 31). Few of our patients had complications related to CNB or SSB procedures, with one patient experiencing mild bleeding that did not result in catheter discontinuation, another experiencing ventricular tachycardia that was thought to be related to sedation medications administered during the procedure, and another having a leaky catheter, which is an equipment malfunction rather than a procedural complication. Most patients had platelet counts >50k and absolute neutrophil count (ANC) values >1,000, minimizing bleeding and infection risks, respectively. The absence of any infectious complications in our study could be related to the high percentage of tunneled catheter use (75%). When catheters are placed using other techniques, prolonged use can increase the risk of inflammation and infection (32), but when the tunneling technique is used, extended use does not correlate with increased risk of infection (33).

Finally, the importance of collaboration between interdisciplinary teams to ensure the needs of the patient and family are being met is evident. Nearly all patients in this study received consultation from the PC and pain medicine specialists. This is significant because delayed PC involvement is linked to a greater chance of dying in the intensive care unit and more intensive interventions that may contribute to increased suffering during the EoL (34–37). Early involvement of the PC team often results in conversations of advanced care planning, including preferences for location of death, DNR/DNI status, and assessment of symptom management needs (31, 38–40). Most of our patients (85%) had POST orders in place before the DOD, 11 patients (41%) stated their preferred LOD, and 63% died in their preferred LOD. More importantly, for patients whose preference was to be in the outpatient setting, SSB interventions may have helped to achieve more-suitable outpatient regimens for adequate pain control; continuing CNB catheters as part of a patient's pain management regimen was a viable option, as seen in 2 study patients. This observation is highly valuable for clinicians caring for children during the EoL as it supports an intervention that can relieve suffering and allow a death at home, if preferred. More than half of our patients died in a hospital setting. We hypothesize that this could be related to other factors such as complex medical comorbidities, not being candidates for outpatient settings, adverse effects of the advanced cancer diagnosis, and being far from their preferred place of death.

The limitations of this study are those inherent to retrospective studies. First, this was a single-institution study with a relatively small cohort of eligible patients. To our knowledge, this is the largest sample size describing regional pain control interventions at the EoL in pediatric oncology. Additionally, retrospective data collection prohibits the procurement of dynamic information related to pain, so we ascertained pain intensity and relief through objective PS documentation and opioid dosing requirements. Given the overall sample size, intercomparison analyses between the types of blocks performed were not powered accordingly and thus not performed and reported. Lastly, our comparison of opioid doses before and after regional pain intervention excluded the use of sustained-release oral or transdermal opioids. We analyzed PCA-delivered opioids exclusively because it is the most “titratable” component of a pain management regimen and likely reflects the change in opioid need in our clinical practice; catheter placements were unaltered during the remaining pain treatments. One may also argue that the lack of evaluation of non-pharmacological interventions for pain and psychology support represents a limitation of this study. Indeed, in the difficult and complex context of EOL, this type of data may have been a valuable component to reflect the multidisciplinary approach to pain management in view of the bio-psycho-social model of pain.

## Conclusion

Our study highlights the analgesic efficacy and safety of regional interventions for relieving pain-related suffering in children dying with progressive cancer and suggests that interventional approaches remain underutilized in this clinical context. The coordinated efforts of the primary oncology service, pain and PC services, and home hospice agencies enabled the implementation of regional pain interventions and their continuation in the outpatient setting. This collaboration is important to help relieve suffering during EoL care and enable preferred location of death. Future prospective studies to investigate the analgesic efficacy and the safety of CNB and SSB in children at the end of life are necessary.

## Data Availability

The original contributions presented in the study are included in the article/Supplementary Material, further inquiries can be directed to the corresponding author.
